# CaCO_3_/BiO_2−x_/CdS Composite with Rapid Photocatalytic Reduction of Cr(VI) Under Visible Light

**DOI:** 10.3390/nano16060376

**Published:** 2026-03-21

**Authors:** Chao Liu, Chongxue Huang, Chaohao Hu, Dianhui Wang, Yan Zhong, Chengying Tang

**Affiliations:** 1 Guangxi Key Laboratory of Information Materials, School of Materials Science and Engineering, Guilin University of Electronic Technology, Guilin 541004, China; lczs@mails.guet.edu.cn (C.L.);; 2 Guangxi Key Laboratory of Calcium Carbonate Resources Comprehensive Utilization, Hezhou University, Hezhou 542899, China

**Keywords:** CaCO_3_, BiO_2−x_, CdS, photocatalytic Cr(VI) reduction, lattice distortion

## Abstract

CaCO_3_/BiO_2−x_/CdS (CCO/BO/CS) ternary composite photocatalyst was synthesized via a hydrothermal method combined with chemical precipitation, and its performance in the photocatalytic reduction of hexavalent chromium (Cr(VI)) under visible light was systematically investigated. Compared with pure BiO_2−x_, CdS, and binary BiO_2−x_/CdS composites, the CCO/BO/CS system exhibited significantly enhanced Cr(VI) reduction activity. Specifically, the CCO/BO/CS (0.75:1:2 wt) composite achieved a Cr(VI) reduction efficiency of 94.53% within 30 min of visible light irradiation—approximately 94.6 times and 6.1 times higher than those of BiO_2−x_ (1.0%) and CdS (15.52%). Photoelectrochemical and trapping experiments revealed that the enhanced performance stems from improved charge separation, accelerated interfacial electron transfer, and the promotional role of CaCO_3_—likely through lattice distortion—rather than direct photocatalytic participation. This study highlights the innovation of incorporating low-cost, eco-friendly calcium carbonate into semiconductor-based photocatalysts to induce lattice distortion for enhanced charge separation, as an effective strategy for improving the reduction efficiency of Cr(VI).

## 1. Introduction

With the acceleration of industrialization, the use of chromium (Cr) has become increasingly widespread, particularly in the electroplating, leather tanning, and dye manufacturing industries. Cr is extensively employed in various industrial applications owing to its excellent corrosion resistance and high hardness [1]. However, the production processes in these industries generate a large amount of wastewater and solid residues containing Cr, which have emerged as significant sources of environmental contamination. It is well known that hexavalent Cr (Cr(VI)) is especially hazardous due to its high toxicity, solubility, and mobility, enabling it to readily migrate through water and soil and thereby posing serious risks to both ecosystems and human health [2]. The direct discharge of untreated industrial effluents containing Cr(VI) leads to severe pollution of soil and groundwater, adversely affecting agricultural productivity, animal survival, and public health. Consequently, there is an urgent need to develop efficient, sustainable, and environmentally benign technologies for Cr(VI) remediation to safeguard environmental and human health [3]. Although conventional wastewater treatment methods—such as chemical precipitation, flotation, and electrolysis—can partially remove Cr pollutants, they often suffer from drawbacks including secondary pollution, high operational costs, and limited efficiency [4]. In this context, semiconductor-based photocatalysis has emerged as a promising green technology for environmental remediation. Compared to traditional approaches, photocatalysis offers distinct advantages, including high degradation efficiency, low cost, and the absence of secondary pollutants [5]. Since its discovery in 1972, photocatalytic technology has been extensively investigated and applied in diverse fields, such as organic dye degradation, heavy metal reduction (including Cr(VI) to Cr(III)), and hydrogen production [6,7,8]. In the natural environment, Cr primarily exists in two oxidation states: trivalent Cr (Cr(III)) and Cr(VI). Cr(III) exhibits significantly lower toxicity than Cr(VI) and is an essential trace element in human nutrition at appropriate concentrations (e.g., 50–200 mg/day for adults), although excessive levels can lead to adverse health outcomes [9,10,11,12].

BiO_2−x_ is a typical mixed-valence compound containing Bi in both +3 and +5 oxidation states. It features a three-dimensional layered structure, specifically a hierarchical architecture assembled by hexagonal nanosheets, that facilitates rapid hole transport. In particular, its valence band arises from the hybridization of Bi 6s and O 2p orbitals, which not only elevates the valence band maximum but also narrows the bandgap to approximately 1.5–2 eV, thereby extending the light absorption range into the visible and even near-infrared regions [13]. Additionally, BiO_2−x_ demonstrates high photochemical stability, with minimal photocorrosion and low metal ion leaching (e.g., Bi ions remain stable without significant dissolution during cycles), reducing the risk of secondary pollution [14]. Compared to other bismuth oxides, BiO_2−x_ exhibits enhanced photocatalytic efficiency under broad-spectrum irradiation, including UV, visible, and near-infrared light, due to its high number of oxygen vacancy defect sites that promote charge separation and carrier mobility [15,16,17]. Owing to these favorable electronic and structural properties, BiO_2−x_ has attracted considerable research interest as a photocatalyst for applications such as organic pollutant decomposition, CO_2_ reduction, and water splitting [18]. CdS, as one of the typical metal sulfides, has been regarded as a promising photocatalyst due to the strong visible light absorption capability and relatively low charge carrier recombination rate, allowing photogenerated electrons and holes to persist for longer durations and thus enhancing photocatalytic efficiency [19,20]. However, CdS photocatalyst also suffers from several inherent limitations, including poor chemical stability, severe photocorrosion under illumination, a tendency to aggregate, and suboptimal utilization of the solar spectrum. Consequently, the photocatalytic performance of pristine CdS is significantly constrained [21,22,23].

Currently, calcium carbonate (CaCO_3_) is a low-cost compound with abundant natural availability, excellent chemical stability, and high thermal resistance. It has found widespread applications across various industries and has also been extensively explored in photocatalysis research [24]. CaCO_3_ can enhance photocatalytic activity by increasing the number of surface active sites and accelerating reaction kinetics. Moreover, it is capable of forming composite structures with other photocatalytic materials, thereby improving their structural stability and operational durability [25,26]. Consequently, the strategic incorporation of CaCO_3_ into photocatalytic systems not only significantly boosts photocatalytic performance but also extends the service life and overall efficiency of the materials, offering new opportunities for environmental remediation and energy conversion applications [27].

In this work, BiO_2−x_/CdS composite photocatalysts with varying mass ratios were synthesized via a chemical precipitation method, using BiO_2−x_ as the matrix. The photocatalytic reduction performance toward Cr(VI) of these composites was evaluated under visible light irradiation, and the composition of the compound with the highest activity was identified. To further enhance the photocatalytic performance of the optimal BiO_2−x_/CdS composite, improve its stability and durability, and simultaneously reduce overall material cost, low-cost CaCO_3_ was incorporated to form a ternary CaCO_3_/BiO_2−x_/CdS (denoted as CCO/BO/CS) composite. The corresponding phase structure, micromorphology, and chemical composition were characterized in detail. The photocatalytic reduction of Cr(VI) over these different composite catalysts was systematically investigated. Finally, a plausible photocatalytic mechanism for Cr(VI) reduction over the CCO/BO/CS composite was proposed.

## 2. Experimental Details

### 2.1. Materials

All chemicals, including NaBiO_3_·2H_2_O (≥99.0%), NaOH (≥99.0%), Cd(NO_3_)_2_·4H_2_O (≥99.0%), Na_2_S·9H_2_O (≥98.0%), and CaCO_3_ (≥99.5%, calcite), were of analytical grade and purchased from Sinopharm Chemical Reagent Co., Ltd., Shanghai, China, and used without further purification.

### 2.2. Preparation of BiO_2−x_

BiO_2−x_ was synthesized via a hydrothermal method. Briefly, 1.68 g of NaBiO_3_·2H_2_O was dissolved in 30 mL of deionized water under continuous stirring for 30 min. Subsequently, 20 mL of a 1.6 mol·L^−1^ NaOH solution was added dropwise to the mixture, which was then stirred for an additional 1 h with the final pH = 13.5. The resulting suspension was transferred into a polytetrafluoroethylene (PTFE)-lined stainless-steel autoclave. The autoclave was sealed and heated at 180 °C for 6 h. After cooling to room temperature, the precipitate was collected by filtration, washed thoroughly with deionized water and ethanol, and dried at 80 °C for 12 h to obtain the BiO_2−x_ powder [14].

### 2.3. Preparation of BiO_2−x_/CdS

First, pure CdS was synthesized as follows: 0.77 g of Cd(NO_3_)_2_·4H_2_O and 0.60 g of Na_2_S·9H_2_O were each dissolved separately in 25 mL of deionized water. The Na_2_S·9H_2_O solution was then slowly added dropwise into the Cd(NO_3_)_2_·4H_2_O solution under continuous stirring. The mixture was stirred for 2 h and subsequently allowed to stand. The resulting suspension was vacuum-filtered, and the collected solid was dried at 60 °C to obtain the CdS sample.

For the synthesis of BiO_2−x_/CdS composites, a predetermined amount of pre-synthesized BiO_2−x_ (0.36 g, 0.18 g, 0.12 g, or 0.07 g) was dispersed into the Cd(NO_3_)_2_·4H_2_O solution (0.77 g in 25 mL deionized water) and stirred for 1 h. After an additional 30 min of stirring, the Na_2_S·9H_2_O solution (0.60 g in 25 mL deionized water) was slowly introduced dropwise under continuous agitation. The reaction mixture was then stirred for a further 2 h. The resulting composite suspensions were processed identically to the pure CdS sample—namely, vacuum-filtered and dried at 60 °C—to yield BiO_2−x_/CdS composites with different mass ratios of BiO_2−x_ to CdS. These samples were designated as BiO_2−x_/CdS (1:1 wt), BiO_2−x_/CdS (1:2 wt), BiO_2−x_/CdS (1:3 wt), and BiO_2−x_/CdS (1:4 wt), respectively.

### 2.4. Preparation of CCO/BO/CS

**Figure 1:**
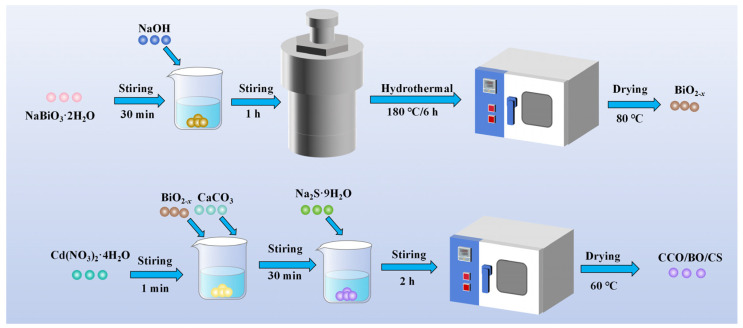
A schematic illustration for preparing CCO/BO/CS composite.

The ternary CCO/BO/CS composites were prepared using the same procedure employed for the BiO_2−x_/CdS (1:2 wt) sample, with the addition of CaCO_3_ during synthesis. Specifically, while dispersing 0.18 g of BiO_2−x_ into the Cd(NO_3_)_2_·4H_2_O solution, predetermined amounts of CaCO_3_ (0.18 g, 0.13 g, 0.09 g, and 0.05 g) were simultaneously introduced. The subsequent steps—including dropwise addition of the Na_2_S·9H_2_O solution, stirring, filtration, and drying at 60 °C—were identical to those described in Section 2.3. The resulting composites were labeled according to their mass ratios of CaCO_3_:BiO_2−x_:CdS. The sample containing 0.18 g of CaCO_3_ (i.e., CaCO_3_:BiO_2−x_:CdS = 1:2:1 by weight) was denoted as CCO/BO/CS (1:2:1 wt), and the remaining samples were labeled analogously based on their respective CaCO_3_ loadings. The synthetic procedure for the CCO/BO/CS photocatalyst was illustrated in Figure 1.

### 2.5. Characterization of Photocatalysts

The crystal phase structures of the as-prepared samples were analyzed by X-ray diffraction (XRD) using a SmartLab diffractometer (Rigaku, Akishima-shi, Japan) with Cu Kα radiation (λ = 1.5406 Å). The morphology and microstructure were examined by scanning electron microscopy (SEM, Quanta 450 FEG, FEI, Hillsboro, OR, USA) and transmission electron microscopy (TEM, Tecnai G2 F30 S-TWIN, FEI, Hillsboro, OR, USA). Surface elemental composition and chemical states were investigated by X-ray photoelectron spectroscopy (XPS) using an ESCALAB 250Xi spectrometer (Thermo Scientific, Waltham, MA, USA), with Al Kα radiation as the X-ray source. UV–Vis diffuse reflectance spectra (UV–Vis DRS) of BiO_2−x_, CdS, BiO_2−x_/CdS, and CCO/BO/CS composites were recorded on a UV-2600 spectrophotometer (Shimadzu, Kyoto, Japan) equipped with an integrating sphere, using BaSO_4_ as the reference standard. Transient photocurrent responses were measured under visible light irradiation from a 300 W xenon lamp (with a 420 nm cutoff filter) using a standard three-electrode electrochemical setup (CHI660E workstation, Chenhua, Shanghai, China). The working electrodes were fabricated by drop-casting dispersions of BiO_2−x_, CdS, or CCO/BO/CS onto fluorine-doped tin oxide conductive glass substrates. The electron paramagnetic resonance (EPR) test used was the EMXplus-6/1 (Bruker, Karlsruhe, Germany). A platinum foil served as the counter electrode, and a saturated calomel electrode was used as the reference electrode. All measurements were carried out in a 0.1 M Na_2_SO_4_ aqueous electrolyte solution. The photoluminescence (PL) spectra were measured by using a fluorescence spectrometer (FS5, Edinburgh Instruments, Livingston, UK).

### 2.6. Method for Evaluating Photocatalytic Reduction of Cr(VI)

Preparation of Cr(VI) solution [28]: A Cr(VI) stock solution was prepared by pipetting 5 mL of the pre-prepared K_2_Cr_2_O_7_ solution (100 mg·L^−1^) into a double-walled quartz reactor, followed by dilution with 95 mL of deionized water to yield a 5 mg·L^−1^ Cr(VI) working solution. Separately, 0.20 g of 1,5-diphenylcarbazide (DPC) was dissolved in a 1:1 (*v*/*v*) mixture of acetone and deionized water (total volume: 100 mL) to prepare the chromogenic reagent. Once fully dissolved, this DPC solution was added to the Cr(VI) solution in the quartz reactor. The pH of the resulting mixture was carefully adjusted to 3.30 ± 0.05 using dilute nitric acid (HNO_3_).

Photocatalytic reduction test: 0.50 g of the photocatalyst was dispersed into the prepared Cr(VI) solution. The resulting suspension was then placed in the dark reaction chamber for 30 min to achieve the adsorption equilibrium. Subsequently, the suspension was irradiated under visible light using a 300 W xenon lamp equipped with a 420 nm cutoff filter (λ > 420 nm), with a light intensity of approximately 100 mW·cm^−2^. A UV–Vis spectrophotometer was baseline-corrected using deionized water, and the concentration of residual Cr(VI) was measured at predetermined irradiation intervals (0, 5, 10, 15, 20, 25, and 30 min) based on the characteristic absorbance of the Cr(VI)–DPC complex at ~540 nm. In addition, cycling stability tests and radical trapping experiments were conducted under identical conditions. For trapping experiments, 1 mmol of a specific scavenger (e.g., EDTA-2Na for h^+^, benzoquinone for •O_2_^−^, or isopropanol for •OH) was added simultaneously with the photocatalyst to the Cr(VI) solution prior to irradiation. The photocatalytic reduction efficiency of Cr(VI) was calculated using the following equation [29]:

(1) $$\eta =\frac{C_{0}-C}{C_{0}}\times 100\%$$

where *η* is the Cr(VI) reduction efficiency (%), *C*_0_ is the initial concentration of Cr(VI) after adsorption equilibrium (i.e., at *t* = 0 min of illumination), and *C* is the Cr(VI) concentration at a given irradiation time.

The photocatalytic process follows the following equation [30]:

(2) $$\ln\left(C_{0}/C\right)=kt$$

Herein, *k* is the apparent pseudo-first-order rate constant.

## 3. Results and Discussion

### 3.1. The Phase Structure and Morphology

**Figure 2:**
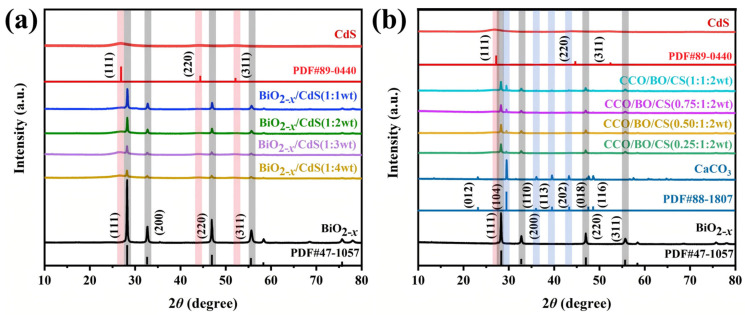
XRD patterns of (**a**) BiO_2−x_/CdS and (**b**) CCO/BO/CS composites with different mass ratios together with those of BiO_2−x_, CdS, and CaCO_3_.

Figure 2 presents the XRD patterns of samples with different composite ratios. The synthesized BiO_2−x_ (JCPDS No.47-1057) and CdS (JCPDS No.89-0440) correspond to the cubic crystalline phases belonging to the space groups of *F*-43*m* and *Fm*-3*m*, respectively. In the XRD patterns of BiO_2−x_/CdS composites with different mass ratios presented in Figure 2a, diffraction peaks attributable to both BiO_2−x_ and CdS are clearly observed. Moreover, as the relative content of BiO_2−x_ decreases, the intensities of its characteristic peaks—corresponding to the (111), (200), (220), and (311) crystallographic planes—gradually decrease, consistent with the reduced phase fraction [31]. Figure 2b displays the XRD patterns of pure BiO_2−x_, CdS, CaCO_3_ (JCPDS No.88-1807), and CCO/BO/CS ternary composites with varying CaCO_3_ loadings. All diffraction peaks in the composite patterns can be unambiguously indexed to the respective single-phase components, with no additional or unassigned peaks detected. Notably, the intensity of the CaCO_3_ peak at 2θ ≈ 29.40° (corresponding to the (104) plane of calcite CaCO_3_) progressively increases with higher CaCO_3_ content, reflecting the greater abundance of this crystalline phase in the composite. Importantly, we noticed that after the addition of CaCO_3_, the diffraction peak (111) of CdS in Figure 2b shifted slightly to the right. This indicates that the introduction of CaCO_3_ may cause some lattice distortion in CdS. However, the inherent crystal structures of CaCO_3_, BiO_2−x_, and CdS did not change significantly overall, and all phases maintained good crystallinity. Furthermore, no foreign diffraction peaks were observed in any of the composite samples, indicating that they have high phase purity. The consistency of peak positions between individual samples and composite samples confirms the successful preparation of cadmium sulfide, BiO_2−x_/CdS, and CCO/BO/CS composite materials, without the formation of secondary phases or solid solutions.

**Figure 3:**
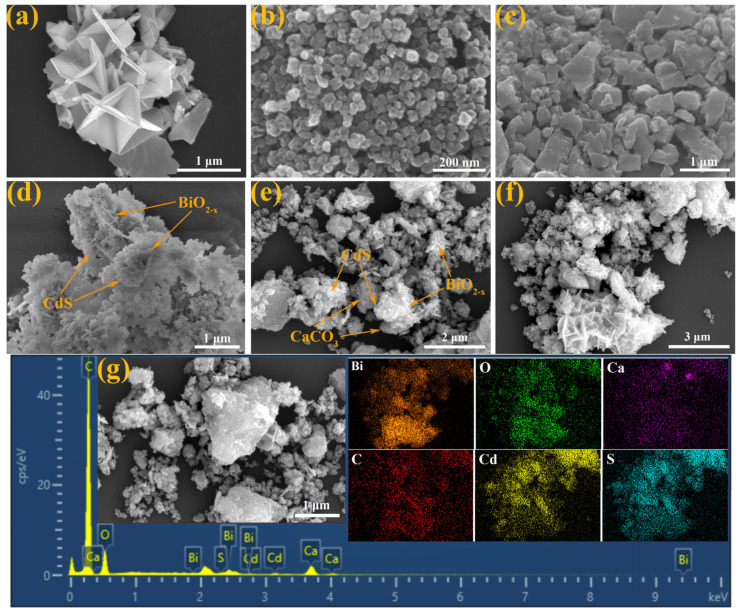
SEM images of (**a**) BiO_2−x_, (**b**) CdS, (**c**) CaCO_3_, (**d**) BiO_2−x_/CdS (1:2 wt), (**e**) CCO/BO/CS (0.75:1:2 wt), and (**f**) corresponding EDS elemental distribution plots show the distribution and elemental composition of Bi, O, Ca, C, Cd, and S. (**g**) CCO/BO/CS (0.75:1:2 wt) after reaction.

Figure 3 shows the SEM images and corresponding EDS results of BiO_2−x_, CdS, CaCO_3_, BiO_2−x_/CdS (1:2 wt), and CCO/BO/CS (0.75:1:2 wt). As shown in Figure 3a, BiO_2−x_ exhibits a clear three-dimensional layered morphology, with individual flakes approximately 0.5–1.5 μm wide, highly aggregated into micron-sized clusters, with no obvious isolated particles and poor dispersibility. In contrast, CdS appears as small and irregular nanoparticles, approximately 30–80 nm in diameter, prone to aggregation (Figure 3b), with moderate dispersibility. CaCO_3_, on the other hand, shows an irregular blocky structure (Figure 3c), approximately 0.2–1.5 μm in diameter, with moderate dispersibility. Through chemical precipitation, cadmium sulfide nanoparticles are uniformly deposited on the surface of the BiO_2−x_ matrix, forming a good BiO_2−x_/CdS (1:2 wt) heterostructure, as shown in Figure 3d, with good dispersibility. In the ternary CCO/BO/CS (0.75:1:2 wt) composite material (Figure 3e), CdS nanoparticles are simultaneously anchored on BiO_2−x_ and CaCO_3_, retaining the distinct morphology of all three independent components with moderate dispersion.

Elemental mapping shown in Figure 3f confirms the homogeneous distribution of Bi, O, Ca, C, Cd, and S throughout the composite, with CdS nanoparticles evenly dispersed across the surfaces of both BiO_2−x_ and CaCO_3_. The coexistence and spatial distribution of all constituent elements provide strong evidence for the successful synthesis of both BiO_2−x_/CdS (1:2 wt) and CCO/BO/CS (0.75:1:2 wt) composites. Moreover, this intimate interfacial contact and well-defined heterostructure are favorable for facilitating the separation and transfer of photogenerated charge carriers, thereby enhancing the overall photocatalytic performance. As shown in Figure 3g, the morphology of CCO/BO/CS (0.75:1:2 wt) after the reaction did not change significantly.

**Figure 4:**
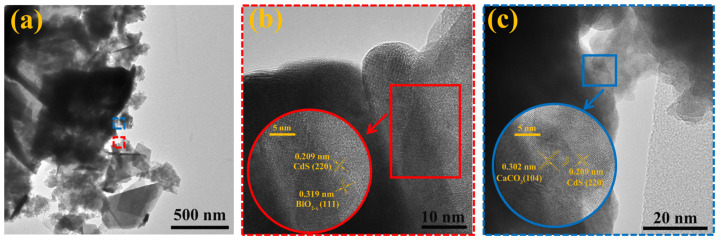
(**a**) TEM and (**b**,**c**) HRTEM images of CCO/BO/CS (0.75:1:2 wt) composite.

To further investigate the microstructure of the CCO/BO/CS (0.75:1:2 wt) composite, TEM and high-resolution TEM (HRTEM) analyses were performed and the measured results were shown in Figure 4. The TEM image in Figure 4a clearly reveals the coexistence of BiO_2−x_, CdS, and CaCO_3_ within the composite. Specifically, the layered structure of BiO_2−x_, the irregular bulk morphology of CaCO3, and the fine nanoparticles of CdS are intimately integrated, forming well-defined and tight interfacial contacts. Such close interfacial junctions are beneficial for efficient interfacial charge transfer among the components [32]. HRTEM images shown in Figure 4b,c display three distinct sets of lattice fringes, corresponding to the individual crystalline phases. The fringe spacing of 0.209 nm is assigned to the (220) plane of cubic CdS. The spacings of 0.319 nm and 0.302 nm match the (111) plane of BiO_2−x_ and the (104) plane of calcite CaCO3, respectively. The stripes show slight curvature or discontinuity at the junctions (small window view in Figure 4c). The clear lattice continuity and coherent alignment at the phase boundaries indicate strong interfacial coupling between the components. These observations confirm the successful fabrication of the ternary CCO/BO/CS (0.75:1:2 wt) heterostructure with well-preserved crystallinity and intimate interfacial contact.

### 3.2. Chemical State Analysis

**Figure 5:**
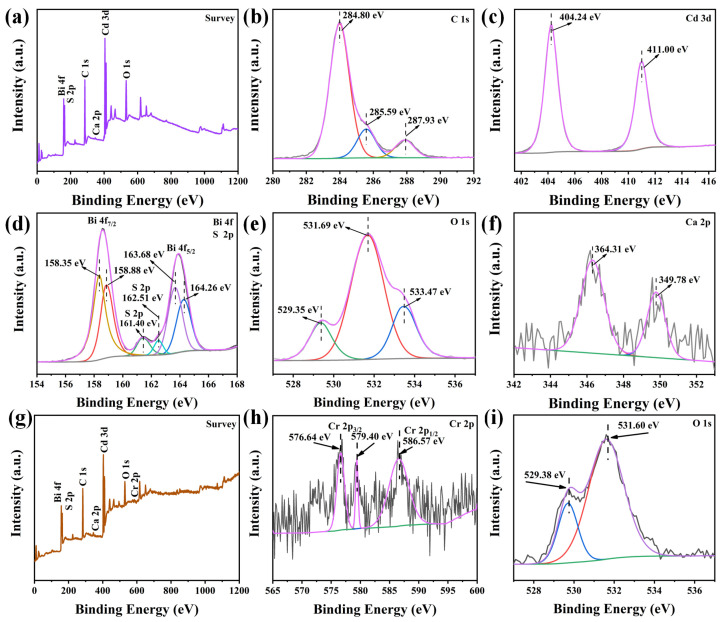
XPS spectra of CCO/BO/CS (0.75:1:2 wt) before reaction: (**a**) survey scan, (**b**) C 1s, (**c**) Cd 3d, (**d**) Bi 4f, (**e**) O 1s, and (**f**) Ca 2p. CCO/BO/CS (0.75:1:2 wt) after reaction: (**g**) survey scan, (**h**) Cr 2p, (**i**) O 1s.

The surface chemical states and elemental composition of the CCO/BO/CS (0.75:1:2 wt) composite before the reaction were investigated by XPS, as shown in Figure 5a–f and Table 1. The survey spectrum in Figure 5a confirms the presence of Bi, S, C, Ca, Cd, and O. High-resolution narrow-scan spectra were acquired for each element. The C 1s spectrum (Figure 5b) can be deconvoluted into three peaks at 284.80 eV, 285.59 eV, and 287.93 eV, corresponding to C–C, C–O–C, and O–C=O bonding configurations, respectively, consistent with the carbonate environment in CaCO_3_ [27]. In the Cd 3d spectrum presented in Figure 5c, two distinct peaks appear at 404.24 eV (Cd 3d_5/2_) and 411.00 eV (Cd 3d_3/2_), with a spin–orbit splitting of ~6.76 eV, being characteristic of Cd^2+^ in CdS [33]. The Bi 4f region in Figure 5d was fitted with two doublets, reflecting the coexistence of Bi in mixed oxidation states. The peaks at 158.35 eV (Bi 4f_7/2_) and 163.68 eV (Bi 4f_5/2_) are assigned to Bi^5+^, while those at 158.88 eV (Bi 4f_7/2_) and 164.26 eV (Bi 4f_5/2_) correspond to Bi^3+^ [34], confirming the presence of both +3 and +5 valence states of bismuth in the composite. The S 2p spectrum (not explicitly labeled but implied in the text) exhibits signals in the binding energy range of 160–163.5 eV, attributable to the S 2p_3/2_ and S 2p_1/2_ components of S^2−^ in CdS [33,35]. The O 1s peak shown in Figure 5e was deconvoluted into three contributions: lattice oxygen (Bi–O) at 529.35 eV, oxygen vacancies at 531.69 eV, and surface-adsorbed oxygen species (e.g., –OH or H_2_O) at 533.47 eV [36], providing clear evidence of oxygen vacancies in the sample. Finally, the Ca 2p spectrum (Figure 5f) shows two symmetric peaks at 349.78 eV (Ca 2p_3/2_) and 364.31 eV (Ca 2p_1/2_), consistent with Ca^2+^ in CaCO_3_. In summary, XPS analysis confirms the successful synthesis of the CCO/BO/CS (0.75:1:2 wt) composite via the chemical precipitation method, with clear evidence of mixed Bi valence states and the presence of oxygen vacancies—features that are beneficial for enhancing photocatalytic activity. Furthermore, the XPS spectra of the CCO/BO/CS (0.75:1:2 wt) composite material after 5 runs of the Cr(VI) reduction reaction were presented in Figure 5g–i for comparison. It can be seen that Cr was detected in the spectrum in Figure 5g. In the Cr 2p spectrum in Figure 5h, 576.64 eV and 579.40 eV are the 2p_3/2_ peaks of Cr(III) and Cr(VI), respectively, while the peak at 586.57 eV is the 2p_1/2_ peak of Cr [37]. This demonstrates that Cr(III) and Cr(VI) adhered to the surface of CCO/BO/CS (0.75:1:2 wt) after the Cr(VI) reduction, which probably reduced the reaction contact area, thus decreasing its efficiency. In Figure 5i, the O 1s peak at around 533.47 eV disappears when compared with the O 1s peak in Figure 5e, indicating that the surface-adsorbed oxygen on the CCO/BO/CS (0.75:1:2 wt) surface is almost completely consumed after the 5 runs of Cr(VI) reduction reaction.

### 3.3. Photocatalytic Activity

**Figure 6:**
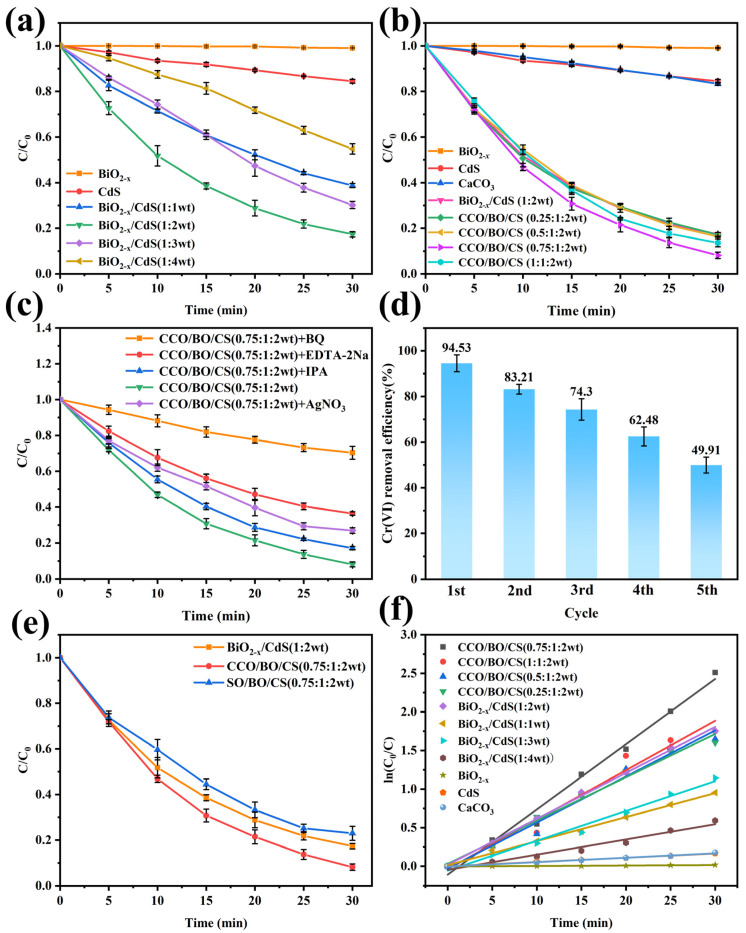
Photocatalytic Cr(VI) reduction performance of (**a**) BiO_2−x_/CdS and (**b**) CCO/BO/CS composites together with BiO_2−x_, CdS, and CaCO_3_ individual components, (**c**) CCO/BO/CS (0.75:1:2 wt) composite affected by different scavengers, and (**d**) cycling stability test of CCO/BO/CS (0.75:1:2 wt), (**e**) control experiments of SiO_2_/BiO_2−x_/CdS (SO/BO/CS) (0.75:1:2 wt), BiO_2−x_/CdS (1:2 wt) and CCO/BO/CS (0.75:1:2 wt), (**f**) the reduction rate constant of Cr(VI) for all tested photocatalysts.

To evaluate the photocatalytic performance of BiO_2−x_, CdS, CaCO_3_, BiO_2−x_/CdS, and CCO/BO/CS composites with varying mass ratios, Cr(VI) reduction experiments were conducted under visible light irradiation (λ > 420 nm), and the measured results were presented in Figure 6, all experiments were performed at least three times independently. As shown in Figure 6a, pure BiO_2−x_ and CdS exhibited limited photocatalytic activity, achieving Cr(VI) reduction efficiencies of only 1.0% and 15.52%, respectively, after 30 min of irradiation. In contrast, all BiO_2−x_/CdS binary composites demonstrated significantly enhanced performance compared to the individual components. Among them, the BiO_2−x_/CdS (1:2 wt) sample displayed the highest activity, achieving a Cr(VI) reduction efficiency of 82.68% within 30 min—approximately 5.33 times higher than that of pure CdS. Given that CaCO_3_ is an abundant, low-cost, non-toxic, and environmentally benign material—especially when compared to BiO_2−x_, CdS, and their precursors—this study further incorporated CaCO_3_ into the optimized BiO_2−x_/CdS (1:2 wt) system to reduce overall catalyst cost and explore potential synergistic effects. As shown in Figure 6b, the incorporation of CaCO_3_ did not suppress the photocatalytic activity of BiO_2−x_/CdS. Instead, at specific compositions, it further enhanced Cr(VI) reduction. Notably, the ternary CCO/BO/CS (0.75:1:2 wt) composite achieved the highest efficiency of 91.87% after 30 min of visible light irradiation, outperforming all other samples. To compare with previous work, we created Table 2 [38,39,40,41] for easy performance comparison.

To identify the dominant reactive species involved in the photocatalytic process, radical trapping experiments were performed using specific scavengers: benzoquinone (BQ) for superoxide radicals (•O_2_^−^), EDTA-2Na for holes (h^+^), AgNO_3_ for photogenerated electrons (e^−^), and isopropanol (IPA) for hydroxyl radicals (•OH) [42,43]. The results presented in Figure 6c indicate that while IPA, AgNO_3_ and EDTA-2Na caused moderate inhibition of Cr(VI) reduction, the addition of BQ led to a dramatic decrease in efficiency—dropping to 29.65%. This strongly suggests that •O_2_^−^ is the primary active species responsible for Cr(VI) reduction in the CCO/BO/CS (0.75:1:2 wt) system. Furthermore, the reusability of the CCO/BO/CS (0.75:1:2 wt) composite was evaluated over five consecutive cycles. As shown in Figure 6d, the Cr(VI) removal efficiency decreased progressively from 94.53% to 49.91% after the fifth cycle. This significant decline of approximately 47% in the Cr(VI) removal efficiency indicates insufficient cyclic stability likely due to factors such as Cr(III) deposition on the catalyst surface, or photocorrosion.

To investigate the indirect promoting effect of CaCO_3_, we added a set of control experiments using SiO_2_/BiO_2−x_/CdS (SO/BO/CS) (0.75:1:2 wt), which was synthesized by the same method as CCO/BO/CS (0.75:1:2 wt). As an inert material, SiO_2_ does not participate in photocatalytic reactions or absorb visible light, so it can serve as a control system for CaCO_3_. Figure 6e shows that the degradation performance of the control sample with SiO_2_ is significantly lower than that of BiO_2−x_/CdS, which confirms the indirect promoting effect of CaCO_3_. In Figure 6f, the reduction rate constant of Cr(VI) can be clearly observed. According to Equation (2), the calculated reduction rate constant (pseudo-first-order reaction rate constant) *k* for CCO/BO/CS (0.75:1:2 wt) is approximately 0.0846 min^−1^, which is 15.32, 142.48, 14.78, and 1.42 times faster than those of CdS, BiO_2−x_, CaCO_3_, and BiO_2−x_/CdS (1:2 wt), respectively.

### 3.4. Possible Photocatalytic Mechanism

**Figure 7:**
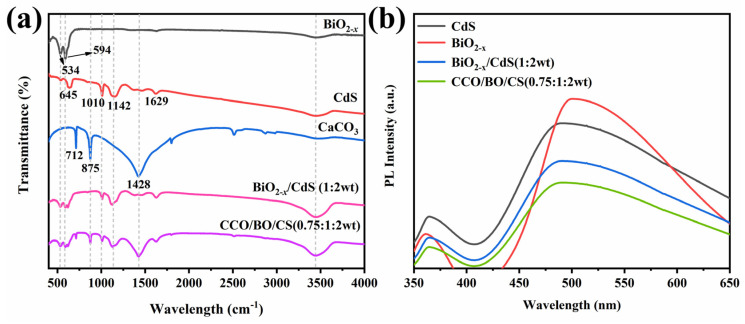
(**a**) FT-IR spectra of BiO_2−x_, CdS, CaCO_3_, BiO_2−x_/CdS (1:2 wt) and CCO/BO/CS (0.75:1:2 wt). (**b**) PL spectra of BiO_2−x_, CdS, BiO_2−x_/CdS (1:2 wt) and CCO/BO/CS (0.75:1:2 wt).

The chemical structure and surface functional groups of the as-prepared materials were analyzed by Fourier-transform infrared (FT-IR) spectroscopy. The measured results shown in Figure 7 indicate that absorption bands at 534 cm^−1^ and 384 cm^−1^ in the FT-IR spectrum of BiO_2−x_ are respectively attributed to the stretching and bending vibrations of Bi–O bonds within the BiO_6_ octahedra [44,45]. For CdS, characteristic absorption bands appear at 645 cm^−1^, 1010 cm^−1^, and 1142 cm^−1^, which are assigned to the bending and stretching vibrations of the Cd–S bond [46,47]. The presence of CO_3_^2−^ groups in CaCO_3_ gives rise to three distinct absorption bands at 712 cm^−1^, 875 cm^−1^, and 1428 cm^−1^ [48]. Specifically, the peak at 712 cm^−1^ corresponds to the in-plane bending vibration, the band at 875 cm^−1^ arises from the out-of-plane bending (deformation) mode, and the strong band at 1428 cm^−1^ is associated with the asymmetric stretching vibration of the carbonate ion [49,50]. Additionally, a broad absorption band centered at 3437.76 cm^−1^ is observed in all samples, which is primarily attributed to the O–H stretching vibrations from adsorbed water molecules on the material surfaces [51]. In the spectrum of the BiO_2−x_/CdS (1:2 wt) composite, characteristic IR bands of both BiO_2−x_ and CdS are clearly present, confirming their coexistence. Upon incorporation of CaCO_3_ to form the CCO/BO/CS (0.75:1:2 wt) ternary composite, additional peaks corresponding to CaCO_3_ emerge, while the original peaks of BiO_2−x_ and CdS remain unchanged in position and shape. This indicates that the addition of CaCO_3_ does not disrupt the intrinsic crystal structures of BiO_2−x_ or CdS. These results confirm the successful integration of BiO_2−x_, CdS, and CaCO_3_ into a three-phase composite without structural degradation or chemical interference. The photoluminescence (PL) spectra in Figure 7b show that BiO_2−x_ exhibits the strongest PL intensity, indicating its highest electron–hole recombination efficiency and consequently the worst photocatalytic performance among the four samples. Compared to the single-phase BiO_2−x_, the formation of a heterojunction in BiO_2−x_/CdS reduces electron–hole recombination, leading to a decrease in its PL intensity. For the CCO/BO/CS composite material, the addition of CaCO_3_ further reduces the electron–hole recombination of BiO_2−x_/CdS, thereby achieving better photocatalytic activity.

**Figure 8:**
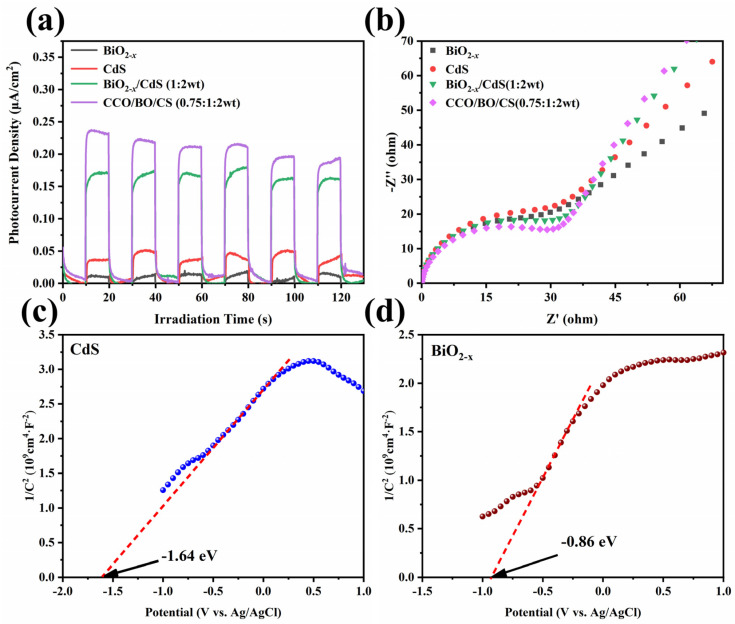
(**a**) Transient photocurrent diagrams and (**b**) EIS Nyquist plots of BiO_2−x_, CdS, CaCO_3_, BiO_2−x_/CdS (1:2wt), and CCO/BO/CS(0.75:1:2 wt). Mott–Schottky test diagrams of (**c**) CdS (**d**) BiO_2−x_.

To further elucidate the behavior of photogenerated charge carriers, transient photocurrent response and electrochemical impedance spectroscopy (EIS) measurements were performed and the results were presented in Figure 8. Figure 8a shows that the BiO_2−x_/CdS (1:2 wt) and CCO/BO/CS (0.75:1:2 wt) composites exhibit significantly enhanced photocurrent responses compared to the individual BiO_2−x_, CdS, and CaCO_3_ components. The photocurrent densities of BiO_2−x_/CdS (1:2 wt) and CCO/BO/CS (0.75:1:2 wt) remain above 0.20 μA·cm^−2^ and 0.17 μA·cm^−2^, respectively, under intermittent visible light irradiation, and demonstrate good stability over multiple on/off cycles. This indicates that the heterojunction formed between BiO_2−x_ and CdS effectively suppresses the recombination of photogenerated electron–hole pairs, thereby enhancing charge separation and ensuring reproducible photoelectrochemical performance [52,53]. Moreover, the incorporation of CaCO_3_ further facilitates interfacial charge transport within the ternary system. The EIS results shown in Figure 8b corroborate this trend: the Nyquist plot of CCO/BO/CS (0.75:1:2 wt) exhibits the smallest arc radius among all tested samples, signifying the lowest charge transfer resistance at the electrode–electrolyte interface. This reduced resistance is markedly lower than that of the single-phase (BiO_2−x_, CdS, CaCO_3_) and binary (BiO_2−x_/CdS) counterparts, indicating significantly accelerated interfacial charge transfer kinetics in the ternary composite [54]. The improved charge separation and transport efficiency directly correlate with the superior photocatalytic activity observed for CCO/BO/CS (0.75:1:2 wt). According to the Mott–Schottky test in Figure 8c,d, we can conclude that both CdS and BiO_2−x_ are n-type semiconductors and their calculated flat-band potential are −1.64 eV and −0.86 eV, respectively.

**Figure 9:**
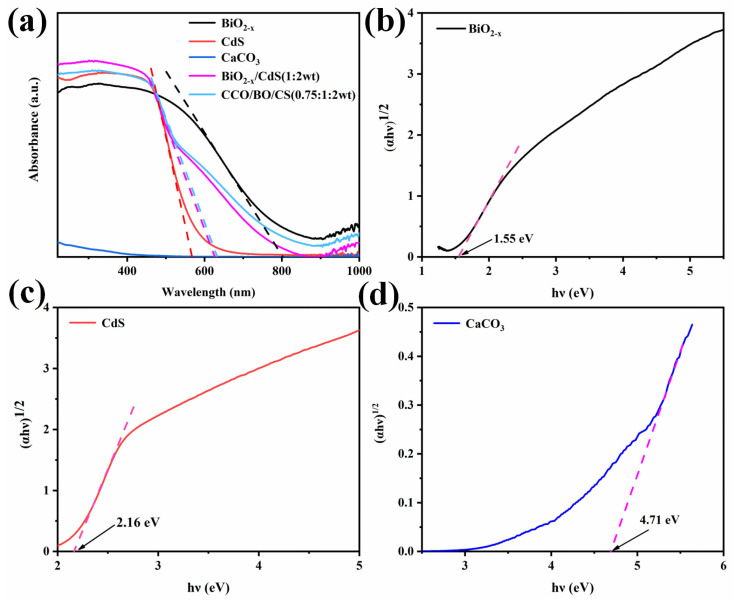
(**a**) UV–Vis DRS spectra and its absorption edge of BiO_2−x_, CdS, CaCO_3_, BiO_2−x_/CdS (1:2 wt), and CCO/BO/CS (0.75:1:2 wt) and Tauc plots for determining the bandgap of (**b**) BiO_2−x_, (**c**) CdS, and (**d**) CaCO_3_.

The optical absorption properties of BiO_2−x_, CdS, CaCO_3_, BiO_2−x_/CdS (1:2 wt), and the CCO/BO/CS (0.75:1:2 wt) composite were investigated by UV–Vis diffuse reflectance spectroscopy (UV–Vis DRS) and the measured results are shown in Figure 9a. The CCO/BO/CS (0.75:1:2 wt) sample exhibits an absorption edge situated between those of BiO_2−x_ and CdS. Notably, compared to pure CdS, the composite displays a pronounced red shift in its absorption onset, thereby extending the light-harvesting range into the visible region. This enhanced visible light absorption improves solar energy utilization and contributes to the superior photocatalytic activity of the ternary composite [55]. The band gap energy (*E_g_*) is calculated by Tauc’s relation [56]:

(3) $${\left(\alpha h\nu \right)}^{1/n}=A\left(h\nu -E_{g}\right)$$

where *α* represents the absorption coefficient, *hν* is the photon energy, *n* depends on the type of semiconductor (direct band gap takes 1/2, indirect band gap takes 2), and *A* is a constant.

The band gap energies of BiO_2−x_, CdS and CaCO_3_ were calculated using the Tauc equation and were found to be 1.55 eV, 2.16 eV and 4.71 eV, respectively. Based on the analysis, the valence band maximum (VBM) of BiO_2−x_ is positioned at +0.96 eV versus the reversible hydrogen electrode (RHE). Using the relationship *E*_g_ = *E*_CB_ − *E*_VB_, the corresponding conduction band minimum (CBM) and VBM positions were determined as follows: CdS: CBM = −0.50 eV, VBM = +1.65 eV; CaCO_3_: CBM = −0.79 eV, VBM = +3.92 eV; BiO_2−x_: CBM = −0.59 eV, VBM = +0.96 eV. Given its wide bandgap of 4.71 eV, CaCO_3_ cannot be photoexcited under visible light irradiation and therefore does not directly generate electron–hole pairs. However, the traditional type II heterojunction or band alignment model (e.g., those proposed by Wang et al. and Cui et al. [57,58]) still applies. This observation is consistent with recent studies on insulator-semiconductor composites. For instance, Hu et al. [59] reported that in BaCO_3_/g-C_3_N_4_ systems, BaCO_3_—an insulator—does not actively participate in photocatalysis but instead induces lattice distortion by incorporating Ba^2+^, thereby promoting charge separation in g-C_3_N_4_. Similarly, Sang et al. [60] proposed that in cadmium sulfide/calcium carbonate composites, small Ca^2+^ ions can partially substitute into the cadmium sulfide lattice, leading to local structural distortions. These distortions form hole traps, which serve as electron trapping sites, thereby improving photocatalytic efficiency. In both cases, the carbonate-based insulator (barium carbonate or calcium carbonate) acts as a structural promoter rather than a direct photocatalytic component. During the preparation of composite photocatalysts, some Ca^2+^ ions with an ionic radius of 1.97 Å in CaCO_3_ can enter the internal unit cell of CdS (diagonal atomic spacing 3.89 Å, adjacent atomic spacing 2.75 Å) [61,62]. The lattice distortions caused by ion entry form many hole traps, which promote the separation of electron–hole pairs on the CdS surface. Meanwhile, due to the potential presence of hole traps on the CdS surface caused by the infiltration of Ca^2+^, and the fact that the conduction band of CdS (−0.50 eV) is more negative than that of BiO_2−x_ (−0.59 eV), some photogenerated electrons from BiO_2−x_ flow to CdS, inhibiting the recombination of electron–hole pairs within BiO_2−x_ and promoting their transport. This explains why the photocatalytic degradation capacity of CCO/BO/CS can be significantly improved.

**Figure 10:**
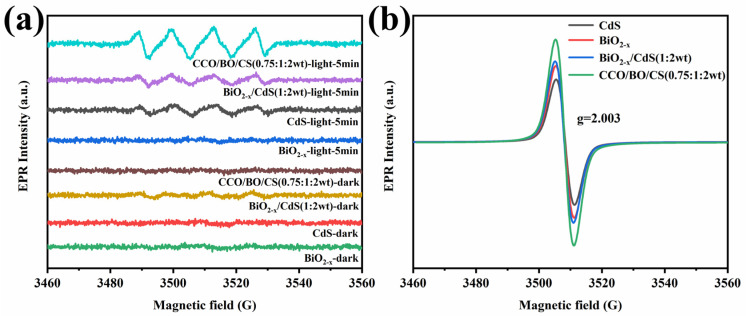
EPR spectra of BiO_2−x_, CdS, CaCO_3_, BiO_2−x_/CdS (1:2 wt), and CCO/BO/CS (0.75:1:2 wt) for (**a**) superoxide radical test and (**b**) vacancy test.

As shown in Figure 10a, the electron paramagnetic resonance (EPR) signal of •O_2_^−^ could not be detected under dark conditions. However, after being exposed to visible light for 5 min, the EPR signal with the characteristics of •O_2_^−^ free radicals could be clearly observed. The results indicate that the CCO/BO/CS ternary composite material can generate •O_2_^−^ free radicals after absorbing visible light, demonstrating strong oxidation and reduction properties [18]. Moreover, after 5 min of illumination, the intensity of the •O_2_^−^ EPR signal detected was significantly higher in CCO/BO/CS compared to BiO_2−x_/CdS and other single phases, indicating that the addition of CaCO_3_ significantly enhanced the generation of •O_2_^−^ free radicals in the BiO_2−x_/CdS heterojunction system, thereby improving the photocatalytic degradation activity of the composite material. Figure 10b shows that there is a significant Lorentzian line-shaped signal at 3460–3560 G and g = 2.003 in the EPR spectrum, which is attributed to the paramagnetic centers formed by oxygen vacancies capturing unpaired electrons in the material [63]. The signal strength relationship of vacancies in different materials is that CCO/BO/CS is stronger than BiO_2−x_/CdS and other single-phase states. In metal oxides, an appropriate amount of oxygen vacancies can capture photogenerated electrons and promote the separation of electron–hole pairs [64,65]. The CCO/BO/CS composite material exhibits the strongest vacancy signal, confirming that the introduction of CaCO_3_ can optimize and stabilize the oxygen vacancy concentration in the composite material.

**Figure 11:**
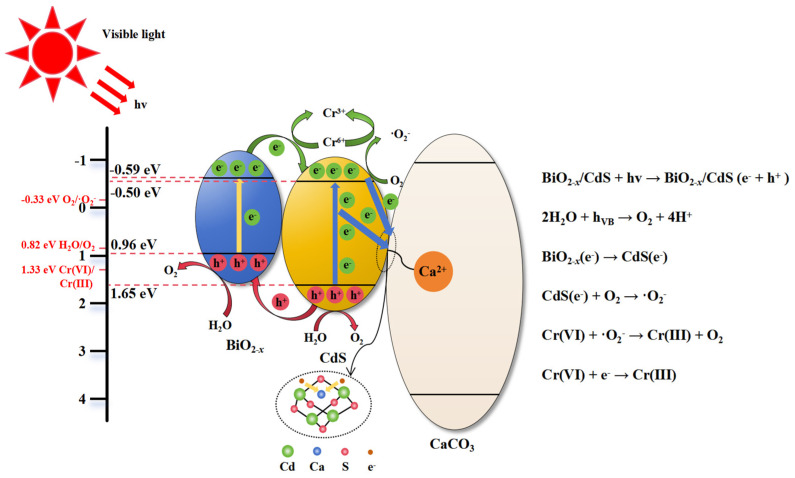
Schematic illustration of the proposed photocatalytic mechanism for Cr(VI) reduction over the CCO/BO/CS composite under visible light irradiation.

Based on the above characterization and photoelectrochemical analyses, a plausible mechanism for the visible light-driven photocatalytic reduction of Cr(VI) over the CCO/BO/CS (0.75:1:2 wt) composite is proposed and illustrated in Figure 11. Under visible light irradiation, both BiO_2−x_ (*E*_g_ = 1.55 eV) and CdS (*E*_g_ = 2.28 eV) absorb photons, generating electron–hole pairs (e^−^/h^+^). The well-matched band alignment between the two semiconductors facilitates efficient interfacial charge transfer. Specifically, the conduction band minimum (CBM) of BiO_2−x_ (−0.59 eV) is slightly more negative than that of CdS (−0.50 eV), while the valence band maximum (VBM) of CdS (+1.65 eV) is more positive than that of BiO_2−x_ (+0.96 eV). This configuration promotes a type-II-like charge transfer pathway: photogenerated electrons in the CB of BiO_2−x_ recombine with holes in the VB of CdS at the interface [66]. Consequently, the most reductive electrons accumulate in the CB of CdS, while the most oxidative holes remain in the VB of BiO_2−x_ [67]. The accumulated electrons in the CB of CdS readily reduce adsorbed O_2_ to generate superoxide radicals (•O_2_^−^), as the CB potential of CdS (−0.50 eV) is more negative than the O_2_/•O_2_^−^ redox potential (−0.33 eV) [29]. As confirmed by the radical trapping experiments (Figure 6c), •O_2_^−^ is the dominant active species responsible for Cr(VI) reduction. The generated •O_2_^−^ (or directly transferred electrons) reduces highly toxic Cr(VI) (Cr_2_O_7_^2−^/Cr^3+^: +1.33 eV) to less toxic Cr(III) [68]. A minor contribution from direct reduction by CB electrons or surface-bound Cr(VI) cannot be excluded. Meanwhile, the oxidative holes retained in the VB of BiO_2−x_ (+0.96 eV) are insufficient to oxidize H_2_O to O_2_ (H_2_O/O_2_: +0.82 eV at pH = 7, but thermodynamically feasible under acidic conditions used in this study, pH ≈ 3.3), though their primary role is to maintain charge balance rather than drive oxidation. Regarding the role of CaCO_3_ in this composite material, we believe that it mainly acts as a structure promoter and lattice distortion inducer and can form hole-trapping sites in cadmium sulfide. Partial incorporation of Ca^2+^ ions into the CdS lattice induces local lattice distortion, which acts as hole-trapping sites and promotes more efficient separation of electron–hole pairs in CdS [60]. Importantly, CaCO_3_ itself—being a wide-bandgap insulator (*E*_g_ = 4.71 eV)—does not participate directly in photoexcitation or redox reactions under visible light but functions as a structural and functional promoter. These interconnected structural features, along with lattice distortion detected by XRD and TEM, oxygen vacancies detected by XPS, enhanced charge separation detected by PL/EIS/photocurrent, and ROS generation detected by EPR, collectively enhance photocatalytic performance, enabling CCO/BO/CS (0.75:1:2 wt) to exhibit excellent photocatalytic degradation performance of Cr(VI).

## 4. Conclusions

In this work, BiO_2−x_/CdS binary and CCO/BO/CS (CaCO_3_/BiO_2−x_/CdS) ternary composite photocatalysts with varying mass ratios were successfully synthesized via a facile chemical precipitation method. Comprehensive characterization techniques confirmed the coexistence of distinct phases, well-defined interfacial structures, and preserved crystallinity in the composites. Under visible light irradiation (λ > 420 nm), the BiO_2−x_/CdS (1:2 wt) composite achieved a Cr(VI) reduction efficiency of 82.68% within 30 min—significantly outperforming the individual BiO_2−x_ and CdS components. To further enhance cost-effectiveness without compromising performance, low-cost and environmentally benign CaCO_3_ was incorporated into the optimized BiO_2−x_/CdS system. The resulting ternary CCO/BO/CS (0.75:1:2 wt) composite exhibited superior photocatalytic activity, achieving a Cr(VI) reduction efficiency of 94.53% under identical conditions. However, the cycling stability test indicates that the reusability of CCO/BO/CS composite needs to be further improved. Photoelectrochemical analyses, including transient photocurrent and electrochemical impedance spectroscopy, revealed that the CCO/BO/CS architecture promotes efficient interfacial charge transfer and suppresses electron–hole recombination, thereby enhancing photocatalytic efficiency. The role of CaCO_3_ is not photocatalytic, but rather structural and promotional—improving light utilization and inducing beneficial lattice distortions that facilitate charge separation. This study demonstrates that the rational design of a low-cost, ternary CCO/BO/CS photocatalyst offers a promising and scalable strategy for efficient Cr(VI) remediation, with significant potential for practical applications in industrial wastewater treatment. Future follow-up studies should systematically investigate key reaction parameters, including solution pH, initial pollutant concentration, and catalyst quality.

## Figures and Tables

**Table 1:** XPS atom composition of CCO/BO/CS (0.75:1:2 wt): (a) before reaction, (b) after reaction.

(a) Before Reaction		(b) After Reaction	
Element	Atomic (%)	Element	Atomic (%)
Bi 4f	1.77	Bi 4f	1.23
C 1s	52.67	C 1s	58.97
Ca 2p	0.42	Ca 2p	0.71
Cd 3d	4.21	Cd 3d	3.81
O 1s	17.88	O 1s	7.97
S 2p	23.05	S 2p	26.96
--	--	Cr 2p	0.34

**Table 2:** A comparison of photocatalytic Cr(VI) reduction performance of several composite catalysts.

Photocatalyst	Dosage (g/L)	Cr(VI) Concentration (mg/L)	Irradiation Time (min)	Degradation Efficiency (%)	Ref.
**CaCO_3_/BiO_2−x_/CdS (This work)**	2.5	2.5	30	91.87	This work
**CdS@MOF@C_3_N_4_**	0.4	20	60	86.8	[38]
**Bi_2_S_3_/Bi_2_MoO_6_**	1.0	50	6	98.5	[39]
**Bi_2_S_3_@g-C_3_N_4_**	0.3	10	120	93.4	[40]
**Fe-doped WO_3_/SiO_2_**	1.0	20	90	91.1	[41]

## Data Availability

Data are contained within the article.
